# Influencing Martensitic Transition in Epitaxial Ni-Mn-Ga-Co Films with Large Angle Grain Boundaries

**DOI:** 10.3390/ma13173674

**Published:** 2020-08-20

**Authors:** Klara Lünser, Anett Diestel, Kornelius Nielsch, Sebastian Fähler

**Affiliations:** 1 Institute for Metallic Materials, Leibniz IFW Dresden, 01069 Dresden, Germany; a.foerster@ifw-dresden.de (A.D.); k.nielsch@ifw-dresden.de (K.N.); s.faehler@ifw-dresden.de (S.F.); 2 Institute of Materials Science, TU Dresden, 01062 Dresden, Germany; 3 Institute of Applied Physics, TU Dresden, 01062 Dresden, Germany

**Keywords:** Ni-Mn-Ga-Co, magnetocaloric effect, hysteresis, epitaxial film, grain boundaries, Heusler alloys, martensitic transition

## Abstract

Magnetocaloric materials based on field-induced first order transformations such as Ni-Mn-Ga-Co are promising for more environmentally friendly cooling. Due to the underlying martensitic transformation, a large hysteresis can occur, which in turn reduces the efficiency of a cooling cycle. Here, we analyse the influence of the film microstructure on the thermal hysteresis and focus especially on large angle grain boundaries. We control the microstructure and grain boundary density by depositing films with local epitaxy on different substrates: Single crystalline MgO(0 0 1), MgO(1 1 0) and Al${}_{2}$O${}_{3}$(0 0 0 1). By combining local electron backscatter diffraction (EBSD) and global texture measurements with thermomagnetic measurements, we correlate a smaller hysteresis with the presence of grain boundaries. In films with grain boundaries, the hysteresis is decreased by about 30% compared to single crystalline films. Nevertheless, a large grain boundary density leads to a broadened transition. To explain this behaviour, we discuss the influence of grain boundaries on the martensitic transformation. While grain boundaries act as nucleation sites, they also lead to different strains in the material, which gives rise to various transition temperatures inside one film. We can show that a thoughtful design of the grain boundary microstructure is an important step to optimize the hysteresis.

## 1. Introduction

Solid state cooling with magnetocaloric materials is a promising way for more efficient and environmentally friendly cooling techniques [1]. The Heusler alloy Ni-Mn-Ga-Co as a possible candidate exhibits a martensitic transition between a high temperature ferromagnetic phase (austenite) and a low temperature phase with lower magnetization (martensite) [2]. However, a major drawback of this first order phase transition is the hysteresis, which can reduce the efficiency of a cooling system drastically. To understand these transition processes in general and the origin of the occurring hysteresis in particular, epitaxial Ni-Mn-based thin films with a defined crystallographic relationship between film and substrate can help [3]. The high surface to volume ratio in thin films allows to examine the martensitic microstructure formation, which proceeds by nucleation and phase growth [4]. Especially the high energy barrier for the martensite nucleation makes a large undercooling necessary and contributes significantly to the transformation hysteresis [3]. Consequently, the hysteresis can be reduced by lowering the energy barriers for the formation of nuclei and their growth. As nucleation barriers are generally lower for heterogeneous nucleation close to defects, this can be achieved through defects induced by ion irradiation [5] or precipitates [6]. Some defects such as nanoindents additionally cause mechanical stress through elastic stray fields, which also helps to reduce the nucleation barrier [7]. Similarly, grain boundaries could act as nucleation sites for the martensitic transition, which was observed in NiTi alloys [8] and calculated for Fe-Pd [9].

Here, we use locally epitaxial films to understand how nucleation and phase growth depend on the film microstructure. For this, we compare the microstructure of Ni-Mn-Ga-Co films grown by DC magnetron sputter deposition on three types of substrates. The term “local epitaxy” describes that the film is not necessarily single crystalline, but may exhibit several, well defined orientation relations to the substrate. This results in large angle grain boundaries. We examine the impact of these grain boundaries on the martensitic transformation, specifically on the transition and hysteresis width. The hysteresis width $\Delta T_{\mathrm{hyst}}$ is typically measured as the difference between the inflection points of the cooling and heating curves, while the transition width $\Delta T_{\mathrm{transition}}$ is determined as the temperature span needed to fully transform the material from austenite to martensite and back. Both temperature intervals are decisive properties for magnetocaloric materials.

## 2. Materials and Methods

Ni-Mn-Ga-Co films with a thickness of 800 nm were prepared by DC magnetron sputter deposition in a UHV chamber (base pressure: $2\times 10^{-9}$ mbar). The films were sputtered in an Ar-2%H atmosphere of $8\times 10^{-3}$ mbar at a deposition temperature of 673 K from an alloyed Ni${}_{44}$Mn${}_{32}$Ga${}_{24}$ and an elemental Co-target. To ensure the same composition of the films, samples were prepared simultaneously on three different, polished substrates from CrysTec: Single crystalline MgO(0 0 1), MgO(1 1 0) and Al${}_{2}$O${}_{3}$(0 0 0 1). Furthermore, the substrate holder was rotated during deposition to achieve a uniform distribution of composition. With energy dispersive X-ray spectroscopy, the film composition was determined to be Ni${}_{45}$Mn${}_{27}$Ga${}_{21}$Co${}_{7}$ with an accuracy below 1 at.%. The film thickness was measured along the film cross section, which was prepared from a reference sample with focused ion beam milling. For pole figure measurements, a four-circle Philips X’pert diffractometer (Philips, Amsterdam, The Netherlands) with CuK${}_{\alpha }$-radiation (λ=0.15406 nm) was used. Electron backscatter diffraction (EBSD) measurements were carried out using a Zeiss LEO 1520 Gemini scanning microscope (Carl Zeiss, Oberkochen, Germany) equipped with a HKL technology Nordlys detector with 20 kV accelerating voltage. A physical properties measurement system (PPMS) with a vibrating sample magnetometry (VSM) option (Quantum Design, Darmstadt, Germany) was used for the thermomagnetic measurements. Additionally, the resistivity of the samples as a function of the temperature was measured in a PPMS in four-point probe geometry. In both cases, the samples were first heated up to 350 K to ensure that they are in an austenitic state and were then cooled down to 50 K and heated up again with a cooling/heating rate of 3 K/min. The constant magnetic field was applied in the film plane for the thermomagnetic measurements and out of plane for the resistivity measurements. Due to a different set-up, the transition temperatures for the thermomagnetic and resistivity measurements differ slightly. We estimate the temperature accuracy to have an experimental error of about 3 K and a systematic error of about 5 K. The transition temperatures ($M_{S}$, $M_{F}$, $A_{S}$ and $A_{F}$) were determined by the intersections of tangents to the curves of the films [3]. The hysteresis width ($\Delta T_{\mathrm{hyst}}$) is calculated from the difference between the inflection points of the heating and cooling curves.

## 3. Results

To probe the influence of different substrate orientations on the film orientation, microstructure and the transition behaviour, we examined samples on three different substrates: MgO(0 0 1) (sample A), MgO(1 1 0) (sample B), and Al${}_{2}$O${}_{3}$(0 0 0 1) (sample C). To exclude that the films differ apart from film orientation and microstructure, we deposited them simultaneously in one run. Consequently, the films were prepared under identical conditions such as sputtering rates and substrate temperatures, and all have the composition of Ni${}_{45}$Mn${}_{27}$Ga${}_{21}$Co${}_{7}$ as measured by EDX. To further prove that the films only differ in the microstructure, we measured the Curie-temperature $T_{C}$ with thermomagnetic measurements (see Supplementary Figure S1 and Table 1). Being an intrinsic property, $T_{C}$ does not depend on the microstructure and should therefore be identical for the three films. Indeed, the values of $T_{C}$ only vary within the measurement accuracy of the device.

**Figure 1:**
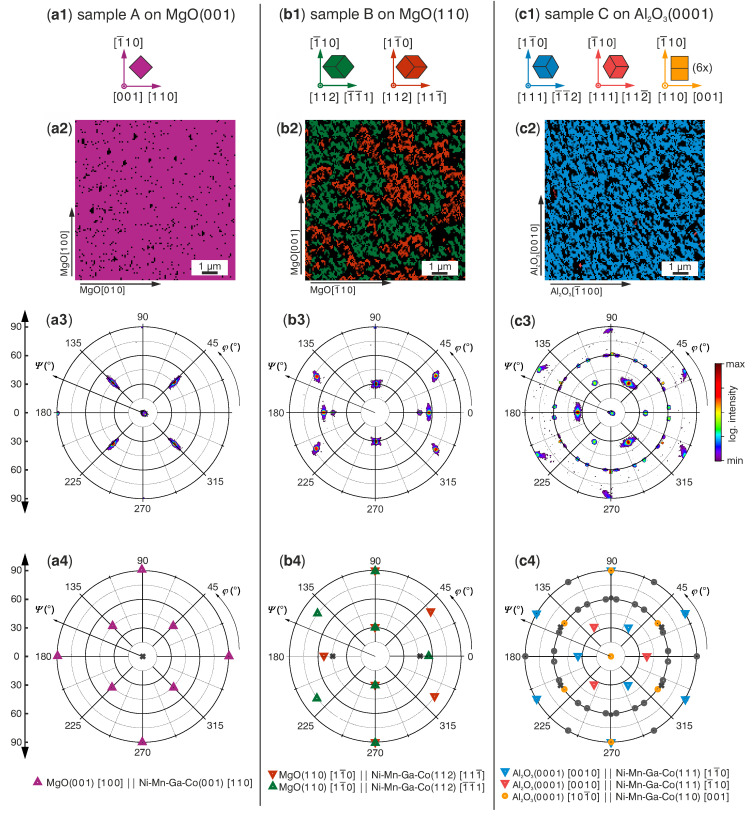
Main orientations and epitaxy relations of the investigated films: First column (**a1**–**a4**): Sample A on MgO(0 0 1), second column (**b1**–**b4**): Sample B on MgO(1 1 0) and third column (**c1**–**c4**): Sample C on Al${}_{2}$O${}_{3}$(0 0 0 1). First row (**a1**–**c1**): Respective sketches of the cubic unit cell in the observed orientation. Second row (**a2**–**c2**): EBSD micrographs displaying the local orientation of representative areas of the films. The colour code refers to the orientations illustrated in (**a1**–**c1**). Black areas refer to points which could not be indexed during measurement. The substrate orientations marked at the edges of these figures are used for all figures in each column. Third row (**a3**–**c3**): {220}-texture measurements in logarithmic scale provide global information of the orientation and are compared with forth row (**a4**–**c4**): Calculated peak positions for the different orientations. The colours of the used symbols correspond to the orientations sketched in (**a1**–**c1**). Peak positions marked with a grey “×” come from the substrates. In (**c4**), only one of the six (1 1 0)-orientations is highlighted in orange for better visibility, the others are sketched in grey and can be obtained by rotating this orientation around [110] in steps of 30${}^{\circ }$.

Figure 1 summarizes the orientations determined by texture measurements and locally by electron backscatter diffraction (EBSD) measurements for all three films. The main orientations are sketched in Figure 1(a1–c1), and the colours are used for all other figures. All samples are austenitic at room temperature, which makes it possible to study the grain orientation without considering the complex martensitic microstructure. Corresponding X-ray diffraction measurements were compared with peak positions from bulk materials [2,10] and can be found in Supplementary Figure S2. All three films grow epitaxially with well defined orientation relations between film and substrate, however, the orientations and microstructures differ as follows. Sample A (Figure 1a, first column) is single crystalline with the (0 0 1) plane parallel to the substrate. With respect to the unit cell of MgO, the unit cell of the austenite is rotated by 45${}^{\circ }$ in plane. This relation was reported for Ni-Mn-Ga-Co films on this substrate before [3]. The roughness of the films was measured with atomic force microscopy (AFM) (see Supplementary Figure S3). With a roughness of $R_{q}\left(A\right)=3.1\;$nm, sample A is smoother than the samples B and C ($R_{q}\left(B\right)=20.3\;$nm and $R_{q}\left(C\right)=18.7\;$nm). As the higher roughness of the samples B and C makes indexing with EBSD more difficult, a higher fraction of points could not be indexed in the EBSD measurements. Still, conclusions can be drawn from the images in combination with the texture measurements. For sample B (Figure 1b, second column) on MgO(1 1 0), the EBSD micrograph shows two kinds of (1 1 2)-oriented grains (red and green). Both orientations grow epitaxially. Their intensity in the pole figure is similar, which means that they occur with equal ratio. The grains are irregularly shaped and their sizes range from 1 μm to about 10 μm. Shape and size distributions of both orientations in the EBSD micrographs are comparable. Additionally, the grains’ shape is anisotropic: Grain dimensions tend to be larger in MgO$\left[\bar{1}\;1\;0\right]$-direction compared to the MgO[0 0 1]-direction. Consisting of differently oriented areas, sample B therefore contains large angle grain boundaries wherever the areas grow together. Sample C on Al${}_{2}$O${}_{3}$(0 0 0 1) (Figure 1c, third column) consists of (1 1 1)- and (1 1 0)-oriented grains with well defined orientations in respect to the substrate. In the EBSD micrograph, however, only (1 1 1)-oriented grains are visible in the studied section of the film. The majority of the examined area belongs to one of the two (1 1 1)-orientation types (blue). Only few grains, smaller than 0.5 μm, can be assigned to the other orientation type (pink). The six different kinds of (1 1 0)-oriented grains are not visible at all in the film section measured with EBSD and appear only in the texture measurements. Indeed, their intensity in the pole figure is two orders of magnitude lower than the intensity of the blue (1 1 1)-orientation type. Therefore, both EBSD and texture measurements confirm that a specific, (1 1 1)-oriented epitaxy relation is favoured under the given deposition conditions. Thus, sample C can be described as mainly single crystalline with a small fraction of differently oriented grains and few grain boundaries. Altogether, the choice of the substrate orientation and material makes it possible to influence both the orientation and the grain boundary density, within the films.

**Figure 2:**
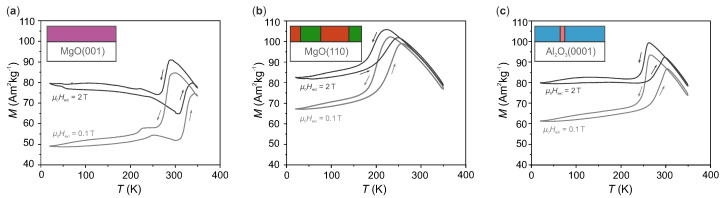
Magnetization measurements as function of temperature at a constant external magnetic field of $\mu_{0}H_{\mathrm{ext}}=0.1$ T and 2 T: (**a**) Sample A on MgO(0 0 1), (**b**) sample B on MgO(1 1 0) and (**c**) sample C on Al${}_{2}$O${}_{3}$(0 0 0 1). Arrows reflect the measurement directions. The sketches in the top left corner of each graph symbolize the microstructure of the films. The colours resemble those of the orientations in Figure 1.

To examine the influence of the different microstructures on the martensitic transition, we compare magnetization measurements in dependence of the temperature for the three films (see Figure 2). For each sample, we measured the magnetization in a low external magnetic field (0.1 T) and a high magnetic field (2 T), which is sufficient to reach saturation magnetization. All curves show a similar trend with distinct differences discussed later. At room temperature and above, the films are austenitic with high magnetization values. When cooling down, the magnetization decreases at the martensitic start temperature ($M_{S}$) as soon as ferromagnetic austenite transforms into martensite with lower magnetization values [2]. The martensitic finish temperature ($M_{F}$) marks the temperature where this decrease is finished. Heating up again, the transition to austenite begins at the austenite start temperature ($A_{S}$) and is completed at the austenite finish temperature ($A_{F}$). Compared to the cooling branch, the magnetization curve for heating is shifted towards higher temperature values. Apart from this hysteresis, the cooling and heating branch are similar. In the higher external magnetic field, the transformation temperatures decrease because the austenite, the phase with higher magnetization, is stabilised. With an external magnetic field of 2 T, the films’ saturation magnetization is reached, whereas magnetic anisotropy is still decisive in 0.1 T. There, the microstructure influences the magnetization change additionally because the magnetic easy axis has a different orientation distribution depending on the general orientation of the film [11]. Not only in low magnetic fields but also in an external magnetic field of 2 T, the magnetization curves for the three films differ with respect to the transition temperatures and the transition intervals.

The different transition behaviours of the films are well visible when plotting the volume fraction of martensite as a function of temperature. Since the magnetization measurements are not only influenced by the martensitic transition, but also by the magnetization change in vicinity to the Curie temperature of the austenite, it is difficult to analyse the fraction of transformed material from the magnetization curves. Therefore, we chose to analyse additional resistivity measurements (see Supplementary Figure S4) to estimate the volume fraction of martensite $f_{\mathrm{martensite}}$ at a certain temperature without considering possible residual austenite at low temperatures. As the resistivity of martensite ($R_{\mathrm{martensite}}$) is significantly higher than the resistivity of austenite ($R_{\mathrm{austenite}}$), we calculate $f_{\mathrm{martensite}}$ as follows:

(1) $$f_{\mathrm{martensite}}\left(T\right)=\frac{R\left(T\right)-R_{\mathrm{austenite}}}{R_{\mathrm{martensite}}-R_{\mathrm{austenite}}}$$

**Figure 3:**
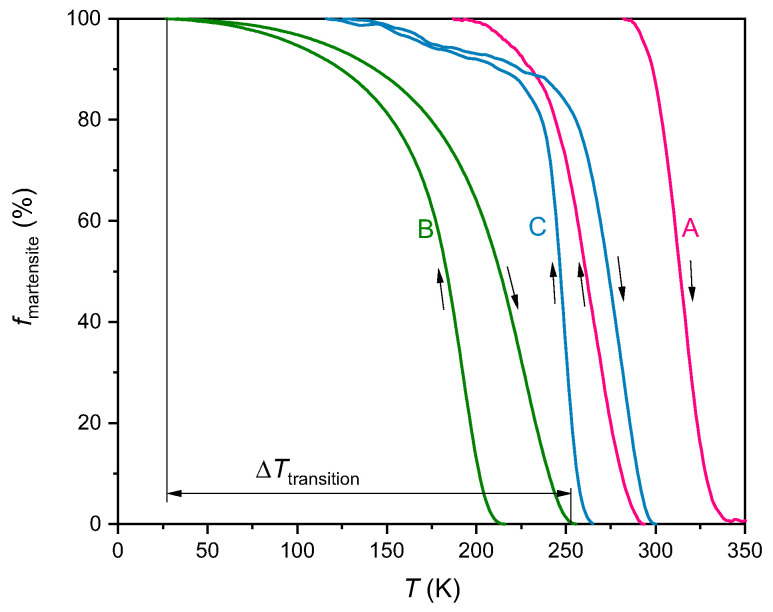
Fraction of martensitic volume $f_{\mathrm{martensite}}$ for sample A on MgO(0 0 1), sample B on MgO(1 1 0) and sample C on Al${}_{2}$O${}_{3}$(0 0 0 1) as a function of temperature for the cooling and heating branch. $f_{\mathrm{martensite}}$ was calculated with Equation (1) from resistivity measurements at a constant external magnetic field of $\mu_{0}H_{\mathrm{ext}}=2$ T. Arrows indicate the measurement directions. The transition width $\Delta T_{\mathrm{transition}}$ is sketched for sample B.

$f_{\mathrm{martensite}}$ was calculated for the heating and cooling curves separately, and $R_{\mathrm{martensite}}$ and $R_{\mathrm{austenite}}$ were defined as the largest and smallest resistivity value of the respective curve. The resulting comparison for all three samples is summarized in Figure 3.

For a discussion of the different transition behaviours of the films, we consider the following characteristic temperature spans: The transition temperature intervals $\Delta T_{\mathrm{mart}}$ and $\Delta T_{\mathrm{aust}}$ are determined from the magnetization measurements by the differences between $M_{S}$ and $M_{F}$, and $A_{S}$ and $A_{F}$, respectively. The hysteresis width $\Delta T_{\mathrm{hyst}}$ was also extracted from the magnetization curves, and it is characterized by the difference between the inflection points of the heating and cooling curves. Moreover, we determined the transition width $\Delta T_{\mathrm{transition}}$ from the resistivity measurements, which is calculated by the temperature difference between 100% volume fraction of martensite on the cooling branch and 0% volume fraction of martensite on the heating branch (see Figure 3). This value describes the temperature interval necessary to transform the whole film from austenite to martensite and back. All characteristic temperatures and temperature spans of the transition are summarized in Table 1.

From the magnetization and resistivity measurements in Figure 2 and Figure 3, we can extract the following differences in the films’ transition behaviour: Firstly, the transition temperatures of the films differ, even though the films were deposited simultaneously and their composition is the same. This can be explained by considering that the films are under different strains. These strains arise due to the misfit strain between substrate and film as well as different thermal expansion coefficients of the substrates. Mechanical strain can lead to changes in the transition temperatures [12,13]. Accordingly, we attribute the differences in transition temperatures in these films to a different stress state. In sample B, the grain boundaries may add local mechanical strain, which could explain the lower transition temperatures for this film [14]. Though our experiments clearly reveal a strong influence of the substrate on the transition temperatures, a detailed analysis of stress is beyond the scope of this paper.

Secondly, the hysteresis width $\Delta T_{\mathrm{hyst}}$ is smaller for the films with grain boundaries than for the film without grain boundaries. Compared to the hysteresis of sample A, the hysteresis is decreased by about 25% for sample B and more than 30% for sample C. This indicates that grain boundaries serve as nucleation sites for the martensite, therefore reduce the nucleation energy barrier and shift the martensitic transition temperatures of the cooling branch closer to the those of the heating branch. This leads, in turn, to a decrease of the hysteresis width. Nevertheless, a reduced hysteresis is not the only effect grain boundaries have on the transition behaviour: Compared to the films without or with only a few grain boundaries (Figure 2a,c), the magnetization changes differently for the film with many grain boundaries (Figure 2b). In particular, the transition temperature intervals $\Delta T_{\mathrm{mart}}$ and $\Delta T_{\mathrm{aust}}$ are considerably larger for sample B than for the other films (compare Table 1). While the magnetization of the samples A and C drops in a small temperature range as soon as $M_{S}$ is reached, the martensitic transition for sample B is more continuous. In addition, the magnetization curve of sample B changes its slope at around 175 K, but the magnetization still decreases when cooling down to 20 K. This two-step transition indicates that the transition is not completed even at low temperatures. The trends from the M(T) measurements can be confirmed with the martensite fraction plotted in Figure 3: The martensite fraction rises gradually in sample B (green curve in Figure 3) and the slope of the curve decreases further towards 100% of martensitic material. The largest transition width of 232 K was also measured for sample B. In contrast, sample A (red curve) transforms at a more constant rate and in a much smaller $\Delta T_{\mathrm{transition}}$ of 155 K. For sample C (blue curve), the martensite fraction reveals a pronounced two-step transition: Around 85% of the material transforms within a small temperature interval of 35 K while the transition of the remaining 15% requires a temperature interval of 121 K.

## 4. Discussion

**Figure 4:**
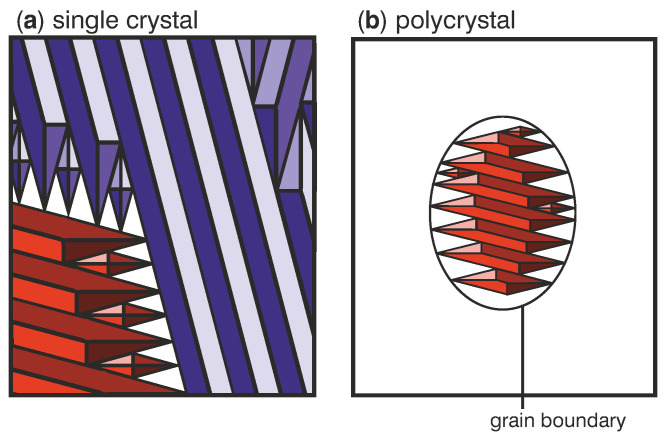
Sketches of the martensitic microstructure in films without grain boundaries (**a**) and with grain boundaries (**b**) illustrate the differences in twinning behaviour. For single crystalline films (**a**), martensitic nuclei can grow into long parallelograms well beyond the depicted area. In contrast, the growth of nuclei in polycrystalline films (**b**) is confined to the inside of the grain. For simplicity, the grain boundary is assumed to be oval and only the microstructure within one grain is shown. (**a**) reprinted from [3] with the permission of AIP Publishing.

In the following, we describe how grain boundaries influence the martensitic transition in general and analyse why the different transition behaviours of sample B and C originate from the presence of large angle grain boundaries. The grain microstructure of the films is formed at high temperatures during deposition when the material is austenitic. This means that the martensitic microstructure has to adapt to the already existing grain boundaries. These large angle grain boundaries are generally incompatible with twin boundaries, a characteristic feature of the martensitic transition, which connect differently oriented martensitic variants. Therefore grain boundaries act as barriers to the formation of a compatible martensitic microstructure. To illustrate the impact of grain boundaries on the martensitic microstructure in films, the transition with grain boundaries (a) and without grain boundaries (b) is sketched in Figure 4. The sketch is based on recent in situ studies, which reveal that the transition proceeds by nucleation and growth of diamonds and parallelograms [4]. An exemplary scanning electron microscopy (SEM) micrograph of an additional polycrystalline Ni-Mn-Ga-Co film exhibiting a martensitic microstructure within grains can be found in Supplementary Figure S5. As sketched in Figure 4a, martensitic variants can grow into long parallelograms in single crystalline films until an interface like the substrate or another martensitic variant is reached. In contrast, the growth of martensitic variants is stopped at the grain boundary in polycrystals (Figure 4b). Close to this grain boundary, untransformed areas of residual austenite remain up to lower temperatures because the martensite can only grow in certain orientations and well defined shapes [4]. In these areas, additional smaller martensitic nuclei are needed to transform the material further. Since small volumes of martensite exhibit a large interface area and thus more twin boundaries per transformed volume, they require a higher total interface energy. As a result, more undercooling is needed to transform smaller austenite volumes. Moreover, the martensite forming in the initial transition causes elastic stress and can make further undercooling necessary [15]. For the transition of a polycrystalline sample, this means that austenite in the middle of the grain transforms to martensite first. In contrast, the austenite close to the grain boundaries remains present at lower temperatures and only transforms during further cooling. Accordingly, upon heating, the martensite close to the grain boundaries already transforms back to austenite at lower temperatures while areas inside of the grain are still stable as martensite. This behaviour is visible as the two-step transition in the magnetization curves (Figure 2b). The same mechanism is also relevant when reducing the grain size, which lowers the transition temperature [16,17]. In smaller grains, the size of individual martensitic variants is limited [18], and the density of interfaces is higher [19]. These effects stabilize the austenite phase and shift the martensitic transition in small grains to lower temperatures. When a variety of grain sizes is present within a film, a wide range of transition temperatures is expected. This will again lead to a broad transition interval.

With this knowledge, we can explain the different transition behaviours of the three Ni-Mn-Ga-Co films: Sample A on MgO(0 0 1) is single crystalline, therefore the transition takes place in a small temperature interval with a uniform temperature dependence and without a two-step transition. In contrast, sample B on MgO(1 1 0) exhibits a large grain boundary density and a large variety of grain sizes ranging from 1 to 10 μm. Both factors lead to the large transition interval and a distinct two-step behaviour. Sample C on Al${}_{2}$O${}_{3}$(0 0 0 1) is mainly single crystalline, therefore the majority of the film transforms in a small temperature interval. The material inside of small grains and in areas close to these grain boundaries transforms only at lower temperatures, thereby creating the observed two-step transition.

For an application in magnetocalorics, not only the hysteresis width but also the transition width for a complete transformation cycle should be as small as possible [20]. Therefore, a microstructure similar to the one in sample C on Al${}_{2}$O${}_{3}$(0 0 0 1) is the most suited, as it leads to a smaller hysteresis and a small transition temperature interval for 85% of transformed material. Possibly, also films with a very uniform grain size and therefore regular spacing of the grain boundaries possess a small hysteresis and transition width. The films presented here can serve as model systems and as a basis to further improve the magnetocaloric properties of Heusler films with a clever microstructural design. An additional post-annealing at different temperatures and annealing times would add further possibilities to influence the grain boundary microstructure.

A broad martensitic transition in Ni-Mn-based films with grain boundaries can be seen in previous publications [21,22,23] and was attributed to substrate constraints or local concentration gradients [24]. Since this effect is not observed for single crystalline films [3,25,26], we propose that it originates from the high density of large angle grain boundaries instead. In bulk polycrystalline materials, grains are usually several hundred micrometer in size and thus larger than the one to ten micrometer large grains in the films discussed here. Consequently, the grain boundary to volume ratio in bulk materials is lower than in thin films and the constraining effect of the grain boundaries is much weaker, which explains why no broad martensitic transition occurs in bulk Heusler alloys.

## 5. Conclusions

To summarize, we presented results of the martensitic transition for epitaxial Ni-Mn-Ga-Co films grown on MgO(0 0 1), MgO(1 1 0) and Al${}_{2}$O${}_{3}$(0 0 0 1) substrates. The choice of substrate allows to tailor the orientation as well as the microstructure of the films, which then influences both the transition temperatures and transition intervals. On the MgO(1 1 0) substrate, the film grew locally epitaxial with large angle grain boundaries and a heterogeneous grain size distribution. In contrast, the sample A on MgO(0 0 1) grew single crystalline without grain boundaries and the sample C on Al${}_{2}$O${}_{3}$(0 0 0 1) mainly single crystalline with very few grain boundaries. By analysing the films with different microstructures, we could show that the hysteresis width $\Delta T_{\mathrm{hyst}}$ is decreased by over 30% for films with large angle grain boundaries compared to single crystalline ones. This indicates that the grain boundaries serve as nucleation sites and facilitate the nucleation of martensite by reducing the energy barrier. However, if the grain boundaries density becomes too large, the transition temperature intervals $\Delta T_{\mathrm{mart}}$ and $\Delta T_{\mathrm{aust}}$ as well as the transition width of the whole transformation cycle $\Delta T_{\mathrm{transition}}$ broaden as well. This makes the films unsuitable for magnetocaloric applications. To explain the enlarged transition width, we discussed the martensitic transition in polycrystalline materials and emphasized that the martensitic microstructure has to adapt to the incompatible grain boundaries. In areas close to the grain boundaries and within small grains, a higher total interface energy is needed for the formation of the martensitic microstructure. This makes a large undercooling necessary. As a polycrystalline film does not transform uniformly, the transition occurs in larger temperature intervals. Our findings indicate that further microstructural design is needed to obtain both a small hysteresis and a narrow transition width to transform the whole film, and we propose to focus on optimizing the grain size.

## Figures and Tables

**Table 1:** Transition temperatures ($M_{S}$, $M_{F}$, $A_{S}$ and $A_{F}$), transition temperature intervals ($\Delta T_{\mathrm{mart}}=M_{S}-M_{F}$ and $\Delta T_{\mathrm{aust}}=A_{F}-A_{S}$) and hysteresis width $\Delta T_{\mathrm{hyst}}$ obtained by M(T) measurements at $\mu_{0}H_{\mathrm{ext}}=2$ T (see Figure 2) and Curie temperature $T_{C}$ from measurements at $\mu_{0}H_{\mathrm{ext}}=0.1$ T (see Supplementary Figure S1). All temperatures and temperature spans are given in K. The transition temperatures were determined by the intersections of the curve tangents. The transition width $\Delta T_{\mathrm{transition}}$ represents the temperature span between 100% volume fraction of martensite on the cooling branch and 0% volume fraction of martensite on the heating branch; it was determined from resistivity measurements.

	Transition Temperatures (K)								
Sample	$M_{S}$	$M_{F}$	$A_{S}$	$A_{F}$	$\Delta T_{\mathrm{mart}}$	$\Delta T_{\mathrm{aust}}$	$\Delta T_{\mathrm{hyst}}$	$T_{\mathrm{transition}}$	$T_{C}$
A on MgO(0 0 1)	276	252	303	324	24	21	49	155	443
B on MgO(1 1 0)	224	186	200	257	38	57	37	232	440
C on Al${}_{2}$O${}_{3}$(0 0 0 1)	256	242	262	294	14	32	33	191	441

## Supplementary Materials

The following are available online at https://www.mdpi.com/1996-1944/13/17/3674/s1, Figure S1: Measurement of Curie temperature with thermomagnetic measurements of Ni-Mn-Ga-Co samples, Figure S2: XRD measurements of Ni-Mn-Ga-Co samples, Figure S3: AFM micrographs of Ni-Mn-Ga-Co samples, Figure S4: *R*(*T*) measurements of Ni-Mn-Ga-Co samples, Figure S5: SEM micrograph of additional, polycrystalline Ni-Mn-Ga-Co film showing the martensitic microstructure.
