# Supplementary material for: Influencing Martensitic Transition in Epitaxial Ni-Mn-Ga-Co Films with Large Angle Grain Boundaries
Source: Materials (Basel). 2020 Aug 20;13(17):3674. doi: 10.3390/ma13173674 (PMC7504521; doi:10.3390/ma13173674)
Supplement: Supplementary file 1 [file materials-13-03674-s001.pdf]

Supplementary Materials: Influencing martensitic transition in epitaxial Ni-Mn-Ga-Co films with large angle grain boundaries

Klara Lünser [1,2,*], Anett Diestel [1], Kornelius Nielsch [1,2,3] and Sebastian Fähler [1,3]

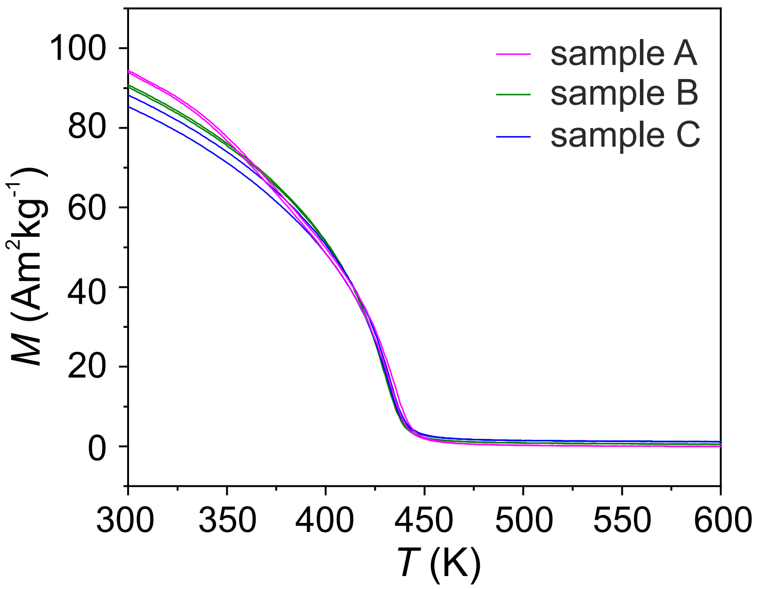

Figure S1. Magnetization in dependence of temperature at a constant external magnetic field of $\mu_0 H_{\mathrm{ext}} = 0.1\,\mathrm{T}$. The Curie temperature was measured at the intersection of tangents to the curves and can be found in Table 1 in the main paper.

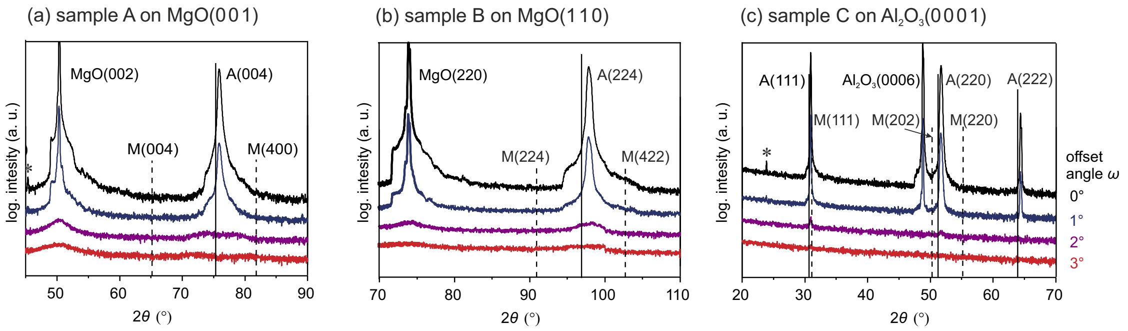

Figure S2. XRD measurements in Bragg-Brentano geometry (radiation: CoK_α) of samples A-C on (a) MgO(0 0 1), (b) MgO(1 1 0) and (c) Al_2O_3(0 0 0 1) showing the substrate peaks and the austenite phase at room temperature measured at offset angles ω from 0° to 3°. Positions of the austenite are labelled with A (continuous lines) [1] and theoretical martensite positions with M (dashed lines) [2]. Peaks marked with asterisks result from the XRD set-up.

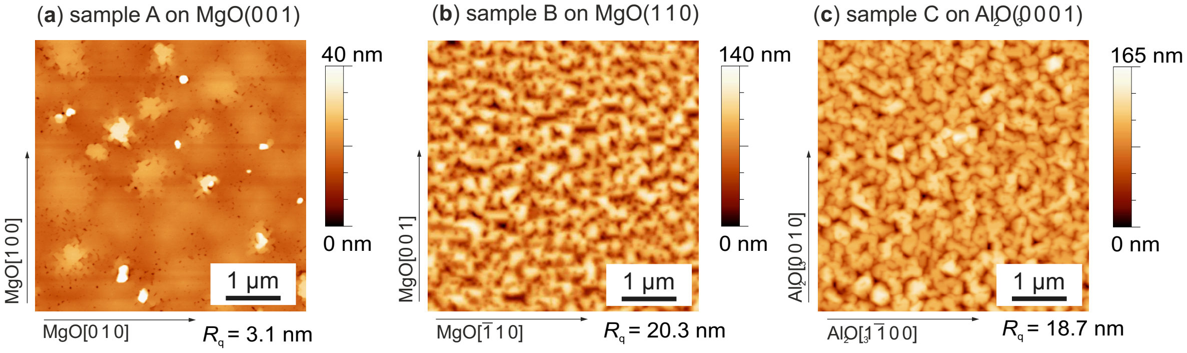

Figure S3. AFM images of Ni-Mn-Ga-Co films: (a) sample A on MgO(0 0 1) has a very smooth surface, (b) sample B on MgO(1 1 0) grows more irregularly with 0.2 to 0.5 μm large features in plane (c) sample C on Al_2O_3(0 0 0 1) has irregular, 0.5 to 3 μm large features laterally without any texture. The z-scale is different for all three samples. The roughness values Rq were determined from 20 × 20 μm^2 areas with an error of around 0.5 nm; they are given below the micrographs.

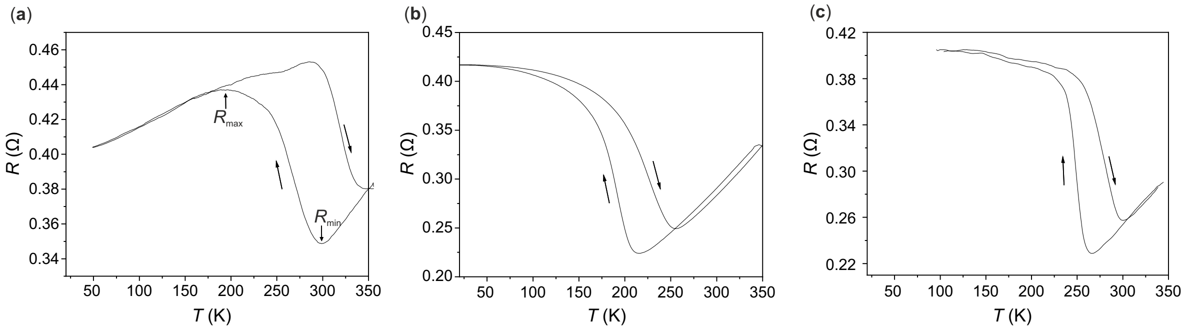

Figure S4. $R(T)$ measurements of samples A-C on (a) MgO(0 0 1), (b) MgO(1 1 0) and (c) Al_2O_3(0 0 0 1) at a constant external magnetic field of $\mu_0 H_{ext} = 2\,T$ show the transition behaviour of Ni-Mn-Ga-Co films. As an example, R_{max} and R_{min} are shown in (a) for the cooling branch. The curves are inverted compared to the $M(T)$ curves because the resistivity of the martensite is higher than the resistivity of the austenite. Apart from that, trends are similar to the $M(T)$ curves. Slight differences in transition temperatures derive from the different set-ups for both measurements. Arrows mark the measurement directions.

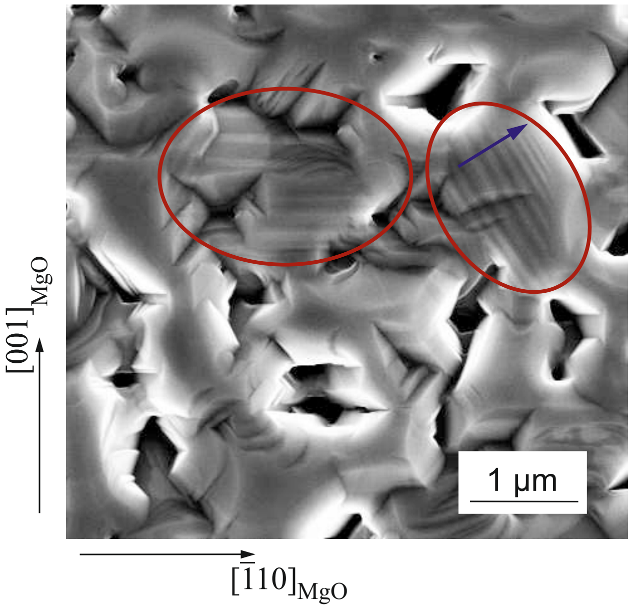

Figure S5. SEM micrograph of a $Ni_{50}Mn_{18}Ga_{23}Co_9$ film on MgO(1 1 0) deposited at 600 °C. Due to the higher deposition temperature the film is martensitic at room temperature, but exhibits large holes. The micrograph shows the characteristic periodically twinned martensitic microstructure in two adjoining grains (circled in red). Following the blue arrow in the right grain, the periodicity of the twinning becomes finer close to the edge of the grain.

References

1. Fabbrici, S.; Albertini, F.; Paoluzi, A.; Bolzoni, F.; Cabassi, R.; Solzi, M.; Righi, L.; Calestani, G. Reverse magnetostructural transformation in Co-doped NiMnGa multifunctional alloys. *Applied Physics Letters* **2009**, *95*, 022508. doi:10.1063/1.3179551.
2. Pons, J.; Chernenko, V.; Santamarta, R.; Cesari, E. Crystal structure of martensitic phases in Ni-Mn-Ga shape memory alloys. *Acta Materialia* **2000**, *48*, 3027. doi:10.1016/S1359-6454(00)00130-0.
